# Research Trends in Octopus Biological Studies

**DOI:** 10.3390/ani11061808

**Published:** 2021-06-17

**Authors:** Anna Di Cosmo, Claudia Pinelli, Anna Scandurra, Massimo Aria, Biagio D’Aniello

**Affiliations:** 1 Department of Biology, University of Naples Federico II, via Cinthia, 80126 Naples, Italy; anna.scandurra@unina.it (A.S.); biagio.daniello@unina.it (B.D.); 2 Department of Environmental, Biological and Pharmaceutical Sciences & Technologies, University of Campania “Luigi Vanvitelli”, 81100 Caserta, Italy; claudia.pinelli@unicampania.it; 3 Department of Economics and Statistics, University of Naples Federico II, via Cinthia, 80126 Naples, Italy; massimo.aria@unina.it

**Keywords:** cephalopods, model species, bibliometrix, bibliometric analysis, science mapping

## Abstract

**Simple Summary:**

Octopuses represent model studies for different fields of scientific inquiry. We provide a bibliometric analysis on biological research trends in octopuses studies by using bibliometrix, a new and powerful R-tool. The analysis was executed from January 1985 to December 2020 including scientific products reported in Web of Science (WoS) database. The main results showed an increasing effort in research involving octopuses with a greater number of journals reporting research on these animals, as well as countries, institutions, and researchers involved. Some research themes lost importance over time, while some new themes appeared recently. Current data provide significant insight into the evolving trends in octopuses studies.

**Abstract:**

Octopuses represent interesting model studies for different fields of scientific inquiry. The present study provides a bibliometric analysis on research trends in octopuses biological studies. The analysis was executed from January 1985 to December 2020 including scientific products reported in the Web of Science database. The period of study was split into two blocks (“earlier period” (EP): 1985−2010; “recent period” (RP): 2011−2020) to analyze the evolution of the research topics over time. All publications of interest were identified by using the following query: ((AK = octopus) OR (AB = octopus) OR (TI = octopus)). Data information was converted into an R-data frame using bibliometrix. Octopuses studies appeared in 360 different sources in EP, while they increased to 408 in RP. Sixty countries contributed to the octopuses studies in the EP, while they were 78 in the RP. The number of affiliations also increased between EP and RP, with 835 research centers involved in the EP and 1399 in the RP. In the EP 5 clusters (i.e., “growth and nutrition”, “pollution impact”, “morphology”, “neurobiology”, “biochemistry”) were represented in a thematic map, according to their centrality and density ranking. In the RP the analysis identified 4 clusters (i.e., “growth and nutrition”, “ecology”, “pollution impact”, “genes, behavior, and brain evolution”). The UK with Ireland, and the USA with Canada shared the highest number of publications in the EP, while in the RP, Spain and Portugal were the leading countries. The current data provide significant insight into the evolving trends in octopuses studies.

## 1. Introduction

The octopuses and their close relatives, cuttlefishes, squids, and nautiloids are representatives of Cephalopods, with around 800 living species described to date [1]. Cephalopods possess interesting biological characteristics such as short lifespan, rapid growth, developmental plasticity, large brain, and sophisticated sense organs with the capacity for learning and memory, which are associated with the ability of rapid adaptation to environmental challenges (both natural and anthropogenic), and made them a resilient group, which may benefit from a changing ocean environment. Therefore, their global sharp increase is not surprising in terms of population in the context of rapid environmental changes in the last six decades [2]. Cephalopods are both important predators and prey in many marine environments and important fishery resources in many countries [3], which together with their peculiar biological features, attracted scientific interest making them optimal models for studies in several different fields of scientific inquiries [4]. Indeed, since the first half of the 20th century, cephalopods also started to play a pivotal role in scientific research, with a steadily growing body of research dealing with various aspects of their biology, including genetics, aquaculture, welfare, behavior, cognition, neurobiology, neuroethology, and the effects of climate change (see [4,5,6,7,8,9,10]).

Among cephalopods, interesting “model organisms” are octopuses. They include about 300 species belonging to several genera [1]. Octopuses have an exceptionally large, well-developed brain (the largest brain-body mass ratio among invertebrates), and flexible high-order cognitive behaviors (e.g., tool use, problem-solving), with a high degree of brain plasticity, learning, and memory [5,11,12,13]. They are active predators, owing to their peculiar sensorial system [14], which could be the evolutionary force-drive behind the development of their unique “abilities” [15]. The combination of very interesting features has fueled an increasing interest by the researchers making them an ideal “model species” not only for inferring brain functioning but also to gain general biological insight and understanding [16]. To date, a worldwide trend in scientific research on octopuses has never been explored using a bibliometric approach [17], while the evaluation of scientific research has become increasingly important in recent years. The bibliometric analysis is a useful tool for measuring the output of scientific research, using specific indicators to obtain information about trends in different fields [18,19].

The present study aims to provide a bibliometric analysis of biological research related to sources, countries, and affiliations dealing with research on octopuses. Furthermore, the structure of the topic was defined both at a conceptual level, by analyzing the co-occurrence network and thematic maps, and at the social structure level, through the analysis of collaboration networks and world maps. The most frequent and impactful journals, countries, research institutes, and their social and conceptual relationship were also identified. We provide a bibliometric analysis of the collection of scientific studies available to these authors related to octopuses from 1985 to 2020 (i.e., 36 years). The period was split into two blocks, comprising roughly the same number of scientific products, to analyze the evolution of the research topics over time. In this work, we provide data helping a wide range of users, not only scientists but also editors, in the choice of topics of emerging and major interest in octopuses studies.

The comprehensive science mapping analysis was performed by using Bibliometrix, a new and powerful R-tool [20] that offers various options for importing bibliographic data from scientific databases and performing bibliometrics analysis related to different items.

## 2. Materials and Methods

### 2.1. Selection Strategy

The present analysis was conducted following the Preferred Reporting Items for Systematic Reviews and Meta-Analysis (PRISMA) guidelines, which illustrates the outcomes of the literature searches and the article selection process [21]. According to our academic license to WoS collection, the bibliometric analysis was executed from January 1985 to December 2020 including scientific products reported in Web of Science (WoS) database, which is now maintained by Clarivate Analytics, as well as articles from the Science Citation Index Expanded (SCI expanded) and the Social Science Citation Index (SSCI). We did not consider other databases, such as Zoological Records and BIOSIS, because of differences in the structure of the records for some fields and relative metadata. The data were collected in March 2021.

The workflow was similar to our recent paper on dog cognition and behavior [22]. All publications of interest were identified by using the following query requiring to search the word “octopus” in the author keywords (AK), in the abstract (AB), and in the title (TI): ((AK = octopus) OR (AB = octopus) OR (TI = octopus)). The system returned 5201 papers. Then, we refined our search selecting only the following WoS categories: Marine Freshwater Biology, Zoology, Genetics Heredity, Fisheries, Biochemistry Molecular Biology, Anatomy Morphology, Neurosciences, Ecology, Biology, Oceanography, Multidisciplinary Sciences, Physiology, Reproductive Biology, Food Science Technology, Cell Biology, Psychology, Environmental Sciences, Psychology Experimental, Behavioral Sciences, Endocrinology Metabolism, Evolutionary Biology, Biodiversity Conservation, Psychology Multidisciplinary, Psychology Biological, Agriculture Dairy Animal Science. This procedure reduced the number of papers to 3295. The next refinement was to select only articles and reviews in English resulting in 2958 papers and to manually eliminate some papers (178) on auditory perception in mammals that had been included for a commonality of terms (“octopus cells”). Then, we obtained our “final collection” of 2780 items.

Data information retrieved by WoS in BibTeX format was exported into Microsoft Excel 2017.

### 2.2. Data Loading and Converting

We loaded the data of our final collection and converted it into an R-data frame using bibliometrix [20], one of the most relevant software tools that contain an extensive set of techniques and it is suitable for practitioners through Biblioshiny [23].

The final collection of the articles was analyzed using different aggregation levels offered by bibliometrix. We divided the periods by the median number of publications to compare a quite similar number of items.

To compare the researcher effort between the two sub-periods, we calculated the Annual Publication Rate per Author as the average of the annual number of publications from the first appearance for each author. The analysis was performed using R own routines applied on bibliometrix output.

Regarding the sources, bibliometrix provides many indicators, such as the number of publications, h-index [24], g-index [25], m-index [26], and the total number of citations received from the articles.

Co-occurrence networks, collaboration networks, thematic maps, and world maps were also generated. A network is a graphical representation of items (terms extracted from article keyword lists, titles, or abstracts) occurring in a set of documents. In a collaboration network, the items consist of the co-authors, author affiliations, or author’s country. A thematic map is a Cartesian representation of the term clusters identified by performing a cluster analysis on a co-occurrence network. It allows an easier interpretation of the research themes developed in a framework. Finally, a world map is a geographical representation of an author’s country network of collaboration. The analyses were based on KeyWords Plus (KWP), which are the words or phrases that frequently appear in the titles of the references cited in an article but do not appear in the title of the article itself. The process to generate the KWP is unique to the Clarivate Analytics databases. A statistical algorithm based on a supervised machine learning approach automatically assigns a set of keywords (i.e., KWP) from a standardized glossary defined for subject categories by a team of experts, extracting them from the documents. Thus, the article’s bibliography is used to identify the research topics and then to label the document with a set of KWP. Using KWP offers several advantages over other databases and the author’s chosen keyword list, covering a broader and more unbiased knowledge base than the author’s subjectivity when providing keywords for their articles [27]. Based on KWP, we have thus obtained the co-occurrence networks, which identify the relationship between the keywords.

The clusters identified by the co-occurrence network were plotted as bubbles in a thematic map graph according to Callon’s centrality and Callon’s density rank values along the two axes [28]. The bubble size is proportional to the occurrences of the words in the cluster. The *X*-axis represents the centrality, that is, the degree of interaction of a network cluster to other clusters appearing in the same graph. It can be considered as a measure of the importance of a theme in the development of the research field. The *Y*-axis symbolizes density, a measure of the internal strength of a cluster network, and can be assumed as a measure of the theme development [29,30,31]. Thus, by the graphical representation of themes we identified: (i) motor themes (first quadrant): the cluster network is characterized by high centrality and high density, meaning that themes are well developed and fundamental for the structuring of a research field; (ii) highly developed and isolated themes (second quadrant): themes characterized by high density and low centrality, meaning that they are of limited importance for the field; (iii) emerging or declining themes (third quadrant): themes with low centrality and low density, meaning that they are weakly developed and marginal. A longitudinal analysis through a thematic evolution allows identifying the emerging or declining trends of a theme [20]. It is achieved by dividing the timespan into different time slices and identifying the trajectory. A direction toward the top right of the map over time identifies an emerging trend, while a direction toward the lower left quadrant would identify a declining trend. (iv) Basic and transversal themes (fourth quadrant): they are characterized by high centrality and low density. It means they are important concerning general topics that are transversal to different research areas of the field.

The social structure of the field was assessed by analyzing the scientific collaboration through the application of the social network analysis [32], applying it at an aggregate level (i.e., countries).

## 3. Results

**Figure 1:**
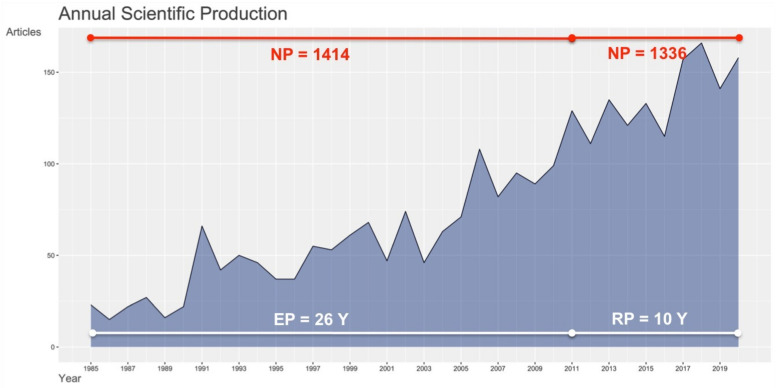
Annual scientific production related to studies on *Octopus*. EP: “earlier period”; NP: number of publications; RP: “recent period”; Y: years.

After our selection, 1414 papers related to studies on octopuses were retrieved in the “earlier period” (EP) (1985–2010), over 26 years, while they were 1366 in the “recent period” (RP) (2011–2020), for 10 years, with an annual growth rate of 6.01% in EP, 3.71% in RP, and 2.28% in the whole period (i.e., 1985–2020) (see Figure 1).

According to the median number of documents, we identified two periods, with 2011 as the cutting edge. Therefore, we analyzed a “recent period” (RP), which extends up to 2020 enclosed (i.e., 10 years), and an “earlier period” (EP), which covered, in the backward direction, a different number of years with respect to PR (i.e., 26 years), running from 2010 to 1985. The number of sources, average citations per year per document, references, KWP, authors’ keywords, authors, author appearances, authors of multi-authored documents, co-authors per documents, and collaboration index showed a positive variation concerning RP, while a negative variation was observed for average citations per document, authors of single-authored documents, single-authored documents, and documents per author, and annual publication rate per author (see Table 1).

The non-overlappping 95% confidence intervals of the annual publication rate per author show a significant difference in researcher efforts between the two sub-periods [EP: 0.290–0.316, RP: 0.254–0.276].

### 3.1. Sources Impact

Octopuses studies appeared in 360 different sources in EP (see Table S1) while they increased to 408 in RP (see Table S2).

In EP, the first three sources with the highest number of h-index were “Marine Biology”, “Aquaculture” and “Marine Ecology Progress Series”. “Marine Biology” and “Aquaculture” had also the highest number of publications, followed by “Marine Ecology Progress Series” and “Fisheries Research”.

According to g-index, “Marine Biology” maintained the first position, while the second source was “Journal of Experimental Biology” and the third was “Aquaculture”. The m-index ranked “Aquaculture” in the first position, followed by “Marine Biology” and “ICES Journal of Marine Science”.

A turnover of sources was observed in the RP, where “PlosOne”, “Science” and “Nature” were the first three sources according to the number of citations (see Table S2). “PlosOne” was the first ranked also according to h-index, g-index, and m-index. “Frontiers in Physiology” produced the highest number of publications, followed by “PlosOne” and “Aquaculture Research”. The second source according to h-index was “Aquaculture” followed by “Journal of Experimental Marine Biology and Ecology”. The g-index saw “Journal of Experimental Marine Biology and Ecology” in the second position and “Aquaculture” in the third position, while “Frontiers in Physiology” and “Aquaculture” were the second and the third sources according to the m-index.

Information about the first ten sources ordered according to the h-index, both for EP and RP, are reported in Table 2. The complete lists for both periods can be found in the Supplementary Material (Tables S1 and S2).

### 3.2. Country Productivity and Affiliations

According to our collection of metadata, 60 countries contributed to the octopuses studies in the EP, while they were 78 in the RP.

In the EP, USA, Japan, and Italy were among the most productive countries. However, looking at the incidence (percentage) of works published by corresponding authors for each country with respect to the total number of works published by a country, Spain ranks first, followed by the USA and Germany.

The leading RP countries were almost the same as in the EP, except for Italy, which lost a few positions and was replaced by China in the third position. China, Japan, and Italy were in the first three positions considering the percentage of publications related to the corresponding authors. Information about the first ten most productive countries ordered according to the number of papers, as well as the number of papers by corresponding authors is reported in Table 3 and Table 4. The whole countries’ productivity data and the countries’ productivity by corresponding authors are given as Supplementary Material (Tables S3–S6).

The number of affiliations also increased between EP and RP, with 835 research centers involved in the EP and 1399 in the RP. The Annual Collaboration Rate per Affiliation also increased from 0.119 [95% CI: 0.106–0.131] in EP to 0.267 in RP [95% CI: 0.239–0.294].

The University of Texas (USA) provided the largest contribution to octopuses research in the EP, followed by the University of Tasmania (Australia) and the University of Aberdeen (UK). This ranking changed substantially in the RP, with the National Autonomous University of Mexico as the main contributor, followed by the Spanish Institute of Oceanography (Spain) and the Ocean University of China. Information about the first ten most productive affiliations ordered according to the number of papers for both periods is reported in Table 5. The complete list can be found in Supplementary Material (Tables S7 and S8).

### 3.3. Conceptual Structure

**Figure 2:**
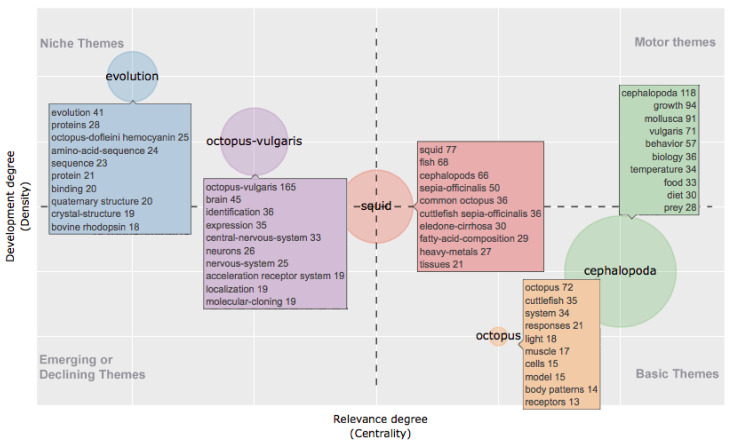
Thematic map showing clusters and the KeyWords Plus from 1985 to 2010 (“earlier period”) identified by the co-occurrence network. The *X*-axis represents the centrality (i.e., the degree of interaction of a network cluster in comparison with other clusters) and gives information about the importance of a theme. The *Y*-axis symbolizes the density (i.e., measures the internal strength of a cluster network, and it can be assumed as a measure of the theme’s development). Accordingly, the first quadrant identifies motor themes (i.e., well developed and important themes for the structuring of a research field); in the second quadrant are plotted highly developed and isolated themes (i.e., themes of limited importance for the field); the third quadrant contains emerging or declining themes (i.e., themes weakly developed and marginal); in the fourth quadrant fall basic and transversal themes (i.e., they concerns general topics that are transversal to different research areas of the field).

The KWP analysis in the EP identified five clusters, represented in a thematic map, according to their centrality and density ranking (Figure 2).

**Figure 3:**
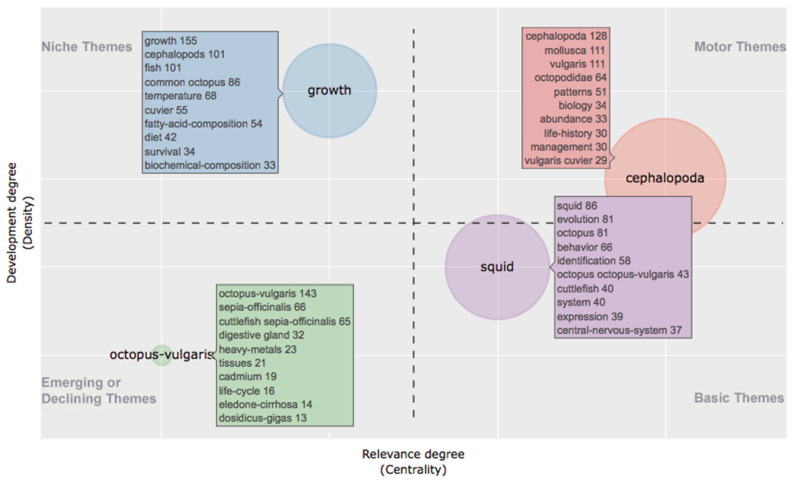
Thematic map showing clusters and the KeyWords Plus from 2011 to 2020 (“recent period”) identified by the co-occurrence network. The *X*-axis represents the centrality (i.e., the degree of interaction of a network cluster in comparison with other clusters) and gives information about the importance of a theme. The *Y*-axis symbolizes the density (i.e., measures the internal strength of a cluster network, and it can be assumed as a measure of the theme’s development). Accordingly, the first quadrant identifies motor themes (i.e., they are well developed and important for the structuring of a research field); in the second quadrant are plotted highly developed and isolated themes (i.e., they are of limited importance for the field); the third quadrant contains emerging or declining themes (i.e., they are weakly developed and marginal); the fourth quadrant includes basic and transversal themes (i.e., themes concerning general topics transversal to different research areas of the field).

In this period there were no motor themes, while two basic themes located in the fourth quadrant showed high centrality and average density. One of them included “cephalopoda”, “growth” and “molluca” as the most co-occurring KWP, and also “food”, “diet” and “prey” which draw a research theme in “*growth and nutrition*”. The other theme had as indicative KWP “system”, “muscle”, “cells”, “body patterns” and “receptors” which are related to “*morphology*”. At the center of the graph there was a cluster characterized by taxonomic terms, but also containing KWP such as “fatty-acid-composition”, “heavy-metals” and “tissues” suggesting a topic related to “*pollution impact*” studied comparatively in different taxa. Two niche themes, with low centrality and high density, were located in the second quadrant. One of them was linked to “*biochemistry*” as it contained the KWP “proteins”, “amino-acid-sequence”, “sequence”. However, the presence of “evolution” as relevant KWP indicates that such studies were mainly aimed at modeling evolutionary patterns. The other one identified a theme on “*neurobiology*” and contained “brain”, “central nervous system” and “neurons” as the most occurring KWP. In the RP the KWP analysis identified four clusters as the result of the emergence/decline of some themes, in terms of gain/loss of centrality and density (Figure 3).

One cluster was positioned in the first quadrant as motor themes in octopuses studies characterized by a high centrality and density. The most occurring KWPs were taxonomic terms, further than “biology”, “abundance” and “life-history”, identifying a theme on “*ecology*”. In the second quadrant (niche themes) there was a cluster characterized by the KWP “growth”, “cephalopods”, “fish”, as well as “temperature”, “fatty-acid-composition” and “diet”, drawing a topic on “*growth and nutrition*”. A cluster in the third quadrant was characterized by low centrality and density, which means that it was weakly developed and marginal and included taxonomic terms, and also “heavy-metals”, “tissue”, “cadmium”, identifying a research theme on “*pollution impact*”. The fourth cluster contained “squid”, “evolution” and “octopus” as the most common KWP, and also “behavior”, “identification”, “expression” and “central nervous system”. It appears to delineate a research theme dealing with “*genes, behavior, and evolution*” and was a basic theme.

### 3.4. Social Structure

**Figure 4:**
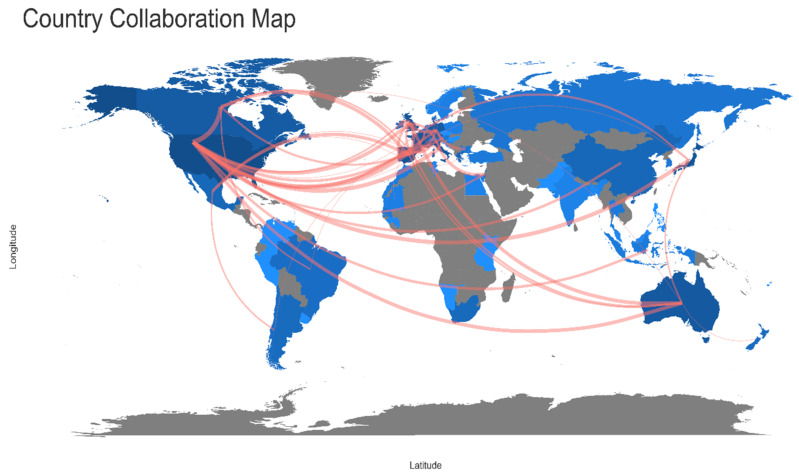
World map showing research collaborations among countries from 1985 to 2010 (“earlier period”). Brighter blue color indicates a higher collaboration rate. Connectors do not show countries with less than three shared papers.

**Figure 5:**
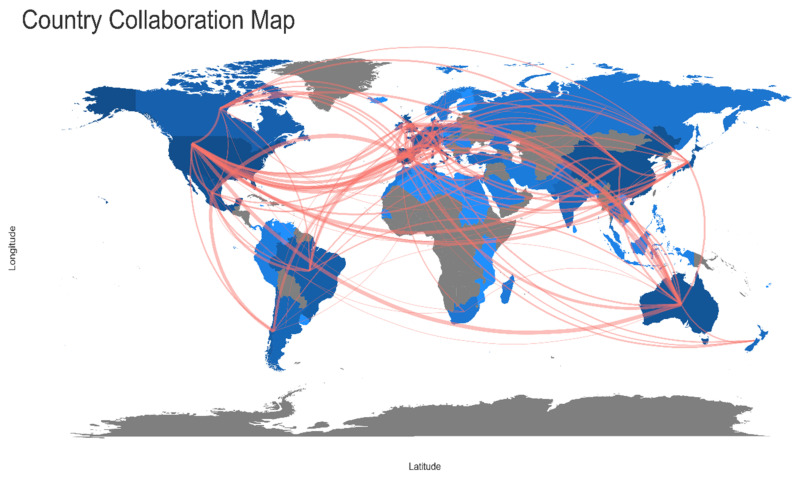
World map showing research collaborations among countries from 2011 to 2020 (“recent period”). Brighter blue color indicates a higher collaboration rate. Connectors do not show countries with less than three shared papers.

The UK with Ireland, USA with Canada shared the higher number of publications, followed by Spain and Portugal and Spain and France in EP. In the RP, after a visible increase in the collaboration network (Figure 4 and Figure 5), Spain with Portugal were the leading countries, with the highest frequency of shared publications. In the second position, Spain showed a higher frequency of collaboration also with the UK and, in the third position, there was the frequency of collaboration between UK and Portugal. Information about the countries that collaborated with a higher frequency for both periods is reported (Table 6). The complete list can be found in Supplementary Material (Tables S9 and S10).

## 4. Discussion

The data relating to the scientific production of studies on octopuses showed a steady increase from the starting point (i.e., 1985) with less than 10 papers per year, to about 100 in the current times and with annual growth of up to 10%. Our bibliometric analysis highlighted a strong increase in the number of authors, author appearances, and authors of multi-authored documents in the more recent period analyzed. Furthermore, in parallel with the increasing number of researchers involved in studies on octopuses, the number of countries and affiliations contributing to the studies has also increased. The statistic “documents per Author” had a negative variation (EP 0.52, RP 0.32), meaning that, while the number of co-authors per publication increased (EP 3.40, RP 5.09), the “annual publication rate per author” decreased (EP 0.30, SP 0.26). In other words, the research effort by each researcher has generally decreased over time. This is not in line with the general trend of scientific studies showing that the contribution of a researchers’ individual publication rate remained unchanged [33]. Despite the decreasing research effort provided by a single researcher over the last decade, the general increase in people (EP 2741, RP 4235) and affiliation collaborations per year (EP 0.119, RP 0.267) can explain the rising trend in studies on octopuses.

Our data show that the most productive countries using octopuses as “model organisms” in different research fields were the same in both periods examined, keeping their contribution almost unchanged. However, some countries, such as Japan, France, and the UK, have shown a decrease in their involvement. The reasons could be the increased commitment of some other countries such as Chile, in the last decade, but also the social and political impact, especially in European countries, due to the recent laws that consider octopuses, along with other cephalopods, “sentience animals” and consequently protected species [34]. Noteworthy, looking at the total number of publications by corresponding authors we obtained a different picture with Spain as the main contributor in the EP, while in the RP no substantial differences emerged with a proportion of corresponding authors around one-third for almost all countries listed in the first ten positions.

Unlike countries, affiliations saw a stronger turnover, with most of the affiliations different in the top ten positions in the two periods considered in our study. The Spanish Institute of Oceanography (Spain) was an important and constant contributor in both periods studied. The University of Texas (USA) and University of Tasmania (Australia), which were listed among the top positions in the EP, have left their position, while emerging affiliations in the last decade, such as the National Autonomous University of Mexico and the Ocean University of China increased their efforts in studying octopuses.

A sharp increase over the past decade has also been observed in the collaboration network, in which the UK, Spain, and Portugal became the leading countries. The general increase in collaboration networks could be beneficial for the dissemination of scientific knowledge at different levels. Articles resulting from international collaborations have been shown to have a higher growth rate than those resulting from national collaborations [35]. Moreover, articles by international co-authors are even more cited [36], which partially explains the increased citation trend of octopuses studies.

In parallel with the rise in research efforts, the number of sources involved in octopuses studies has also increased. Considering the number of citations it is evident that there was a strong turnover comparing the two periods of study and important journals such as “Science” and “Nature” were highly ranked only in the RP. However, they published only a few studies, and indeed they did not receive a high h-index and the other bibliometric indicators. Based on h-index, only a few journals remained high-ranked in both periods, such as “Aquaculture” but others have significantly changed their rankings. Of note, “Marine Biology”, the journal with the highest h-index value in the EP, was not even listed in the top sources in the last decade and “Marine Ecology Progress Series” the journal with the third-highest h-index, was in ninth position in the RP Contrariwise, the “Journal of Experimental Marine Biology and Ecology” gained a higher rank in recent times. It should be noted that the h-index alone cannot be indicative of the relevance of a journal. Moreover, it is noteworthy that some journals were inaugurated after 1985, as in the case of “PLoS One” that was launched in 2006, and it is, therefore, logical that it was only slightly represented in the old period.

According to the methodology for measuring themes development by strategic maps [31] (the research fields as clustered by the KWP), two themes were present in both periods of study, namely “*growth and nutrition*” and “*pollution impact*”. Both were subjected to a decreased interest, considering the Callon centrality and density measures [31]. Particularly, “*pollution impact*” became a declining theme in RP, while “*growth and nutrition*” lost centrality, passing from motor themes in the EP to niche themes in the RP.

In the past decade, “*morphology*”, “*biochemistry*” and “*neurobiology*” themes disappeared, probably partly incorporated in other themes, while “*ecology*” and “*gene, behavior, and brain evolution*” appeared as new themes, the first as a motor theme and the latter as a basic theme. It should be emphasized that this picture comes from the most occurring KWP, which does not exclude the possibility of the existence of other lines of research that, however, not being sufficiently developed did not cluster in a specific theme. Noteworthy, in several clusters, appeared other cephalopod species, indicating that many studies have addressed specific issues using a comparative approach.

A recent review on the state-of-the-art of cephalopods research explored the scientific production in different fields ranging from genetics, aquaculture, climate change, anthropogenic impact, and animal welfare in captivity, also including behavior, cognition, and neurobiology [4]. The authors using the research topics mentioned above as keywords (e.g., “aquaculture”, “behavior”, “cognition”, etc.) associated with the term “cephalopod” have provided the number of publications per decade between 1986 and 2015 as derivative from research on Clarivate Web of Knowledge Core Collection (WoS). In the three decades examined (1986–1995; 1996–2005; 2006–2015), the studies in the field of aquaculture and behavior prevail by far, compared to those of genetics and neuroscience/neurobiology, to which only more recently are associated those relating the climate change, the cognition, and welfare. Our study on octopuses articles seems to provide a somewhat different picture of the research topics addressed, which means that octopuses are used as model studies often in different fields from the other cephalopods or with a different degree of involvement. However, it should be mentioned that O’Brien and collaborators restricted their research to some specific keywords, while our search strategy took into consideration all fields of octopuses studies. This could be another explanation why the octopuses studies do not exactly match with that of the whole group of Cephalopods [37], thus making it difficult to compare with the current data.

It should be emphasized that our algorithm clusters the most frequent KWP, which does not exclude that octopuses are involved in other research. Important themes such as “*genetics*” and “*genomics*” described for Cephalopods [4] appear still poorly developed in octopuses with respect to other themes to be evident in our analysis. However, this does not exclude that this theme could become an emergent theme as proved by recent papers that link genes expression to behavior [5,12].

In this paper only articles from Web of Science (WoS) were considered, thus our data does not cover the entire literature on octopuses (i.e., grey literature). However, this is a general limitation since no scientific database is comprehensive, and each of them has its power and weaknesses [38]. Furthermore, since our academic subscription to the WoS collection did not allow electronic access to researches published before 1985, our study was limited to the last 36 years. However, although the number of items considered in this study might not precisely reflect the worldwide biological research activity on octopuses the current data provide significant insight into the evolving trends in octopuses studies.

## 5. Conclusions

This study, despite the general limitation of scientific databases, provides a bibliometric analysis of octopus research sources, countries, and affiliations that have contributed most over time, as well as a network of co-occurrences and thematic maps. Current data provides meaningful insight into evolving trends in octopus studies by helping a wide range of users, not only scientists but also editors, to understand emerging and most relevant topics.

## Figures and Tables

**Table 1:** Main information about the collection reported separately for the “earlier period” (EP: 1985–2010) and the “recent period” (RP: 2011–2020).

Main Information about Collection		
Timespan	EP (1985–2010)	RP (2011–2020)
**Document Types**		
Total	1414	1366
Article	1348	1278
Review	66	88
**Data**		
Sources	360	408
Average citations per documents	32.26	12.48
Average citations per year per doc	1.66	1.80
References	34,424	47,238
**Document Contents**		
Keywords Plus (ID)	3644	4294
Author’s Keywords (DE)	2807	3813
**Authors**		
Authors	2741	4235
Author Appearances	4859	6951
Authors of single-authored documents	124	52
Authors of multi-authored documents	2617	4183
**Authors Collaboration**		
Single-authored documents	184	58
Documents per Author	0.52	0.32
Annual Publication Rate per Author	0.30	0.26
Co-Authors per Documents	3.44	5.09
Collaboration Index	2.13	3.20

**Table 2:** Information on the first ten sources ordered according to the h-index, from 1985 to 2010 (“earlier period”: EP) and 2011 to 2020 (“recent period”: RP). NP: number of publications; TC: total citations; h-index: journal’s number of published articles (h), each of which has been cited in other papers at least h time; g-index: the largest number such that the top g articles received at least g2 citations; m-index: the ratio h/n, where h is the h-index and n the number of years since the first published paper. PY: publication year. The first three items are in bold.

**Source Impact EP (1985–2010)**							
	**Source**	**TC**	**NP**	**h-index**	**g-index**	**m-index**	**PY_start**
1	Marine Biology	**1944**	**47**	**30**	**43**	**0.81**	1985
2	Aquaculture	**1944**	34	**28**	**34**	**1.00**	1994
3	Marine Ecology Progress Series	**1580**	31	**24**	31	0.67	1986
4	Fisheries Research	**1184**	39	22	33	0.71	1991
5	Journal of Experimental Biology	1434	**42**	21	**37**	0.57	1985
6	Journal of Experimental Marine Biology and Ecology	1207	32	21	32	0.58	1986
7	Bulletin of Marine Science	960	**43**	20	29	0.65	1991
8	Biochemistry	854	25	18	25	0.49	1985
9	Biological Bulletin	882	24	17	24	0.53	1990
10	Journal of Zoology	702	20	17	20	0.47	1986
**Source Impact RP (2011–2020)**							
	**Source**	**TC**	**NP**	**h-index**	**g-index**	**m-index**	**PY_start**
1	Plos One	**968**	**48**	**19**	**29**	**1.73**	2011
2	Aquaculture	469	34	**15**	**20**	**1.36**	2011
3	Journal of Experimental Marine Biology and Ecology	520	29	**14**	**22**	1.27	2011
4	Journal of Experimental Biology	334	23	13	18	1.18	2011
5	Aquaculture Research	411	**44**	12	17	1.09	2011
6	Fisheries Research	382	40	12	16	1.09	2011
7	Current Biology	380	13	12	13	1.09	2011
8	Frontiers in Physiology	411	**54**	11	16	**1.38**	2014
9	Marine Ecology Progress Series	300	20	11	17	1.00	2011
10	General and Comparative Endocrinology	341	13	8	13	0.73	2011

**Table 3:** Information on production of the first ten most productive countries ordered by the total number of publications (freq) from 1985 to 2010 (“earlier period”: EP) and from 2011 to 2020 (“recent period”: RP). The first three items are in bold. C: corresponding author.

Country Production EP (1985–2010)				Country Production RP (2011–2020)			
	Region	Freq	% by C		Region	Freq	% by C
1	**USA**	**443**	**58.2**	1	**USA**	**532**	30.5
2	**Japan**	**279**	47.3	2	**Spain**	**501**	34.3
3	**Italy**	**246**	40.2	3	**China**	**388**	**37.1**
4	Spain	230	**63.5**	4	Mexico	375	28.0
5	United Kingdom	187	56.1	5	Italy	309	**35.6**
6	France	153	43.8	6	Australia	256	31.6
7	Australia	144	55.6	7	United Kingdom	205	27.3
8	Germany	115	**56.5**	8	Portugal	203	29.6
9	Canada	98	48.0	9	Japan	164	**36.6**
10	Portugal	98	44.9	10	Chile	155	21.9

**Table 4:** Information about production of the first ten countries ordered by the total number of publications by corresponding authors (CNP) from 1985 to 2010 (“earlier period”: EP) and from 2011 to 2020 (“recent period”: RP). CFreq: citation frequency according to the publication by the corresponding author; SCP: single country publications (i.e., number of articles in which all authors belong to the same country); MCP: multiple countries publications (i.e., number of articles including at least a co-author working in a different country with respect to the corresponding author); TNP: the number of publications; TC: total citation received; The first three items are in bold.

**Country Production by Corresponding Author EP (1985–2010)**						
	**Country**	**CNP**	**Freq**	**SCP**	**MCP**	**MCP_Ratio**
1	**USA**	**258**	**0.20**	**216**	**42**	0.16
2	**Spain**	**146**	**0.11**	**104**	**42**	0.29
3	**Japan**	**132**	**0.10**	**124**	8	0.06
4	United Kingdom	105	0.08	78	**27**	0.26
5	Italy	99	0.08	76	23	0.23
6	Australia	80	0.06	61	19	0.24
7	France	67	0.05	43	**24**	0.36
8	Germany	65	0.05	55	10	0.15
9	Canada	47	0.04	36	11	0.23
10	Portugal	44	0.03	36	8	0.18
**Country Production by Corresponding Author RP (2011–2020)**						
	**Country**	**CNP**	**Freq**	**SCP**	**MCP**	**MCP_Ratio**
1	**Spain**	**172**	**0.13**	**103**	**69**	0.40
2	**USA**	**162**	**0.12**	**119**	**43**	0.27
3	**China**	**144**	**0.11**	**116**	28	0.19
4	Italy	110	0.08	72	38	0.35
5	Mexico	105	0.08	61	**44**	0.42
6	Australia	81	0.06	44	37	0.46
8	Portugal	60	0.04	34	26	0.43
7	Japan	60	0.04	47	13	0.22
9	United Kingdom	56	0.04	20	36	0.64
10	Brazil	46	0.03	33	13	0.28

**Table 5:** The first ten most productive affiliations from 1985 to 2010 (“earlier period”: EP) and from 2011 to 2020 (“recent period”: RP). The first three items are in bold.

Most Productive Affiliations EP (1985–2010)			Most Productive Affiliations RP (2011–2020)		
	Affiliations	Articles		Affiliations	Articles
1	Univ Texas	49	1	Univ Nacl Autonoma Mexico	103
2	Univ Tasmania	43	2	Inst Espanol Oceanog	93
3	Univ Aberdeen	33	3	Ocean Univ China	54
4	Hebrew Univ Jerusalem	31	4	Univ Aveiro	39
5	Univ Lethbridge	28	5	Hebrew Univ Jerusalem	36
6	Univ Caen	26	6	Shanghai Ocean Univ	36
7	Inst Espanol Oceanog	25	7	Univ Austral Chile	36
8	Univ Padua	25	8	Univ Vigo	35
9	Inst Invest Marinas	23	9	La Trobe Univ	30
10	Univ Nacl Autonoma Mexico	22	10	Univ Tasmania	28

**Table 6:** Information about the countries (first ten positions) with the higher frequency of collaboration from 1985 to 2010 (“earlier period”: EP) and from 2011 to 2020 (“recent period”: RP). The first three items are in bold.

**Collaboration EP (1985–2010)**			
	**From**	**To**	**Frequency**
1	**United Kingdom**	**Ireland**	**16**
2	**USA**	**Canada**	**16**
3	**Spain**	**France**	**12**
4	**Spain**	**Portugal**	**12**
5	**Usa**	**Italy**	**11**
6	**Usa**	**Japan**	**11**
7	Spain	Canada	9
8	Spain	United Kingdom	9
9	United Kingdom	Australia	9
10	USA	Australia	9
**Collaboration RP (2011–2020)**			
	**From**	**To**	**Frequency**
1	**Spain**	**Portugal**	**49**
2	**Spain**	**United Kingdom**	**36**
3	**United Kingdom**	**Portugal**	**31**
4	Spain	Mexico	30
5	Usa	Australia	30
6	Italy	United Kingdom	26
7	Mexico	Chile	25
8	USA	China	22
9	Australia	United Kingdom	21
10	United Kingdom	France	19

## Data Availability

The datasets generated and/or analyzed during the current study are available from the corresponding author on request.

## Supplementary Materials

The following are available online at https://www.mdpi.com/article/10.3390/ani11061808/s1, Table S1: Information about all sources ordered according to the h-index, from 1985 to 2010 (“earlier period”: EP), Table S2: Information about all sources ordered according to the h-index from 2011 to 2020 (“recent period”: RP), Table S3: Information about countries production ordered by the total number of publications from 1985 to 2010 (“earlier period”: EP), Table S4: Information about countries production ordered by the total number of publications from 2011 to 2020 (“recent period”: RP), Table S5: Information about countries production ordered by the total number of publications by corresponding authors (CNP) from 1985 to 2010 (“earlier period”: EP), Table S6: Information about countries production ordered by the total number of publications by corresponding authors (CNP) from 2011 to 2020 (“recent period”: RP), Table S7: The most productive affiliations from 1985 to 2010 (“earlier period”: EP). The first three items are in bold, Table S8: The most productive affiliations from 2011 to 2020 (“recent period”: RP). The first three items are in bold, Table S9: Numbers of shared publications between countries in the period 1985–2010 (“earlier period”: EP), Table S10: Numbers of shared publications between countries in the period 2011–2020 (“recent period”: RP).
